# Evaluation of Wheat Chromosome Translocation Lines for High Temperature Stress Tolerance at Grain Filling Stage

**DOI:** 10.1371/journal.pone.0116620

**Published:** 2015-02-26

**Authors:** Gautam Prasad Pradhan, P. V. Vara Prasad

**Affiliations:** 1 Williston Research Extension Center, North Dakota State University, Williston, North Dakota, United States of America; 2 Department of Agronomy, Kansas State University, Manhattan, Kansas, United States of America; Department of Agriculture and Food Western Australia, AUSTRALIA

## Abstract

High temperature (HT, heat) stress is detrimental to wheat (*Triticum aestivum* L.) production. Wild relatives of bread wheat may offer sources of HT stress tolerance genes because they grow in stressed habitats. Wheat chromosome translocation lines, produced by introgressing small segments of chromosome from wild relatives to bread wheat, were evaluated for tolerance to HT stress during the grain filling stage. Sixteen translocation lines and four wheat cultivars were grown at optimum temperature (OT) of 22/14°C (day/night). Ten days after anthesis, half of the plants were exposed to HT stress of 34/26°C for 16 d, and other half remained at OT. Results showed that HT stress decreased grain yield by 43% compared with OT. Decrease in individual grain weight (by 44%) was the main reason for yield decline at HT. High temperature stress had adverse effects on leaf chlorophyll content and Fv/Fm; and a significant decrease in Fv/Fm was associated with a decline in individual grain weight. Based on the heat response (heat susceptibility indices, HSIs) of physiological and yield traits to each other and to yield HSI, TA5594, TA5617, and TA5088 were highly tolerant and TA5637 and TA5640 were highly susceptible to HT stress. Our results suggest that change in Fv/Fm is a highly useful trait in screening genotypes for HT stress tolerance. This study showed that there is genetic variability among wheat chromosome translocation lines for HT stress tolerance at the grain filling stage and we suggest further screening of a larger set of translocation lines.

## Introduction

High temperature (HT, heat) stress is one of the most important environmental stresses that adversely affects growth, development and yield of many field crops including wheat [1–4]. High temperature stress is a principal wheat yield-decreasing factor in Central Asia, North Africa, Europe, Australia, and the United States [5–7]. A simulation study in Australia showed wheat yield could decrease up to 50% when growing season temperature became 2°C warmer than the average [8]. Wheat is usually planted in late autumn to early winter and harvested before summer; therefore, most of the wheat growing areas of the world experience HT stress at grain filling to ripening stages. The average wheat yield in the U.S. Great Plains is often less than half of the yield obtained in cooler regions of the world because the air temperature usually exceeds 30°C during the grain filling period [9].

High temperature stress at the grain filling stage of wheat (10 d after anthesis to physiological maturity) leads to yield loss because it adversely affects physiological traits and individual grain weight (IGW). The first visual symptom of HT stress in wild and spring wheat is decreased leaf chlorophyll [1,10], which could be attributed to thylakoid membrane damage [11,12], lipid peroxidation of chloroplast membranes [13], and inhibition of chlorophyll biosynthesis [14]. Chlorophyll loss, in turn, is a large contributor to a loss in the photosynthetic capacity of wheat leaves [15]. Reynolds et al. [16] reported a significant correlation between the rate of chlorophyll loss and photosynthetic capacity of flag-leaves at the grain filling period of wheat grown in a hot environment.

High temperature stress overexcites chlorophyll molecules, which leads to formation of excessive production of reactive oxygen species (ROS) such as superoxide anions and hydrogen peroxide, and it decreases the activities of antioxidant enzymes such as superoxide dismutase (SOD) and catalase (CAT). These effects weaken the cellular defense mechanism and result in damaged cellular membranes, lost chlorophyll, and reduced photosynthetic capacity [13,17].

Decreased IGW because of HT stress at the grain filling stage of wheat is a principal cause of yield loss [15,18–20]. Gibson and Paulsen [18] observed a decrease in IGW of 18% to 29% when hard red winter wheat cultivar ‘Karl 92’ was subjected to HT stress of 35/20°C (day/night) from 10 to 20 d after anthesis. Yang et al. [20] reported about a 50% decrease in IGW of synthetic wheat subjected to HT stress of 35/20°C at 10 d after anthesis. The authors of the current study recently observed a 39% average decline in IGW of synthetic hexaploid and bread wheat subjected to HT stress of 36/30°C at 21 d after anthesis [15]. The decrease in IGW under HT stress is attributed to increased leaf senescence and decreased grain-filling duration [8,10,20,21]. High temperature often increases grain-filling rate, but it cannot compensate for the negative effect on grain filling duration [3,22].

The climatic models presented by Meehl and Tebaldi [23] and the Intergovernmental Panel on Climate Change [24] have predicted more frequent and longer-lasting episodes of hot days and nights in wheat growing areas of the world. One of the most efficient ways to obtain sustainable and stable economic yield under a HT stress environment will be to develop stress-tolerant cultivars [25,26]. Development of stress-tolerant cultivars warrants identification of tolerant genotypes (e.g., landraces and wild species), followed by introgression of the tolerant genes from these identified genotypes into modern wheat cultivars. Wild relatives of bread wheat are native to the Mediterranean region and may be sources of HT stress tolerance genes because they grow in stressed habitats. In wheat, genetic variability for abiotic and biotic resistance can be developed through inter-specific and inter-genomic crossings. Inter-specific crossing is accomplished by hybridizing wheat cultivars and landraces, whereas inter-genomic crossing might include crossing between wheat and genome donor species such as *Aegilops speltoides* Tausch (putative B genome donor), *Aegilops tauschii* Coss. (D genome donor), or crossing between wheat and non-genome donor species such as *Secale cereale* L. (R genome), *Agropyron intermedium* (Host) Beauvois (E and X genomes), *Aegilops geniculata* Roth (U^g^M^g^ genome), *Dasypyrum villosa* (V genome), etc. An inter-genomic crossing between wheat and non-genome donor species requires special techniques that are described elsewhere [27]. It has been reported that *Haynaldia villosa* is a good source of genes for resistance to powdery mildew and leaf rust [27], and *Aegilops speltoides* was recently identified as resistant to HT stress at anthesis [1,28]. The wheat-alien chromosome recombinant lines, or wheat chromosome translocation lines (CTLs), developed through inter-genomic crossings, have shown greater tolerance to biotic stress such as powdery mildew, wheat curl mite, and leaf and strip rusts [29–32], but knowledge on performance of chromosome translocation lines under HT stress is limited. Because the translocation lines have chromosome segments from alien species native to stressed habitats, we hypothesize that genetic variability will exist among chromosome translocation lines for tolerance to HT stress. The objectives of this study were to evaluate wheat chromosome translocation lines for tolerance to HT stress at the grain filling stage and identify physiological and yield traits associated with the tolerance.

## Materials and Methods

### Experimental Materials, Crop Husbandry, and Treatments

Sixteen randomly selected CTLs with a small segment of chromosome transferred from mosquito grass [*Dasypyrum villosum* (L.) P. Candargy] to cultivar ‘Chinese Spring’ (CS) (9 lines) [33], from ovate goatgrass [*Aegilops geniculata* Roth] to cultivar TA4305–118 (4 lines) [32], from goatgrass [*Aegilops speltoides* Tausch] to cultivar ‘Chinese Spring’ (1 line) [34], from mammoth wildrye [*Leymus racemosus* (Lam.) Tzvelev] to cultivar ‘Chinese Spring’ (1 line) [35], and from intermediate wheatgrass [*Thinopyrum intermedium* (Host) Barkworth & D.R. Dewey] to cultivar ‘Chinese Spring’ (1 line) [36] were used in this study. Four bread wheat cultivars, ‘Chinese Spring’, ‘WL711’, ‘C306’, and ‘HUW206’, were used as checks (Table 1). ‘Chinese Spring’ and ‘WL711’ were parents to the chromosome translocation lines; whereas, ‘C306’ and ‘HUW206’ were drought tolerant and susceptible wheat cultivars, respectively [37]. All the plant materials were kindly provided by Dr. Bikram S. Gill, Wheat Genetic and Genomic Resources Center, Kansas State University, Manhattan, Kansas.

**Table 1:** List of wheat chromosome translocation lines and cultivars used in the present study.

TA #^†^	Designation of translocation chromosome	Description	References	
TA5594	T4DS·4V#3L	‘Chinese spring’- *Dasypyrum villosum* TL^‡^		[33]
TA5595	T4DL·4V#3S	‘Chinese spring’- *Dasypyrum villosum* TL		[33]
TA5616	T1DL·1V#3S	‘Chinese spring’- *Dasypyrum villosum* TL		[33]
TA5617	T6AS·6V#3L	‘Chinese spring’- *Dasypyrum villosum* TL		[33]
TA5636	T3DL·3V#3S	‘Chinese spring’- *Dasypyrum villosum* TL		[33]
TA5637	T3DS·3V#3L	‘Chinese spring’- *Dasypyrum villosum* TL		[33]
TA5638	T5DL·5V#3S	‘Chinese spring’- *Dasypyrum villosum* TL		[33]
TA5639	T7DL·7V#3S	‘Chinese spring’- *Dasypyrum villosum* TL		[33]
TA5640	T7DS·7V#3L	‘Chinese spring’- *Dasypyrum villosum* TL		[33]
TA5599	T5M^g^S·5M^g^L-5DL	‘WL711’–*Aegilops geniculata* TL		[32]
TA5600	DS5M(5D)	‘WL711’–*Aegilops geniculata* TL		[32]
TA5601	T5DL·5DS-5M^g^S(0.75)	‘WL711’–*Aegilops geniculata* TL		[32]
TA5602	T5DL·5DS-5M^g^S(0.95)	‘WL711’–*Aegilops geniculata* TL		[32]
TA5088	T5DS·5S#3L	‘Chinese spring’- *Aegilops speltoides* TL		[34]
TA5608	T7AL·7Lr#1S	‘Chinese spring’- *Leymus racemosus* TL		[35]
TA5624–4	T7BS·7S#3L	‘Chinese spring’- *Thinopyrum intermedium* TL		[36]
TA3008	-	‘Chinese Spring’		-
TA4350–118	-	‘C306’		-
TA4350–119	-	‘HUW206’		-
TA4305–118	-	‘WL711’		-

^†^Wheat Genetics and Genomics Resource Center collection accession number;

^‡^TL, Translocation Line.

Seeds were sown in 1.6-L plastic pots of dimensions 14 cm (height) × 50 cm (top perimeter) × 36 cm (bottom perimeter) filled with a potting mix (Metro Mix 360; Hummert International, Topeka, KS) and 8 g of Osmocote Plus (N:P_2_O_5_:K_2_O = 15:9:12; Scotts, Marysville, OH), a slow-release fertilizer. Six pots of plants per genotype were randomly divided into three identical growth chambers (two pots per chamber) (Conviron, Winnipeg, MB, Canada) set at an optimum temperature (OT) of 22/14°C day/night, ~85% humidity, 16 h photoperiod, and photosynthetically active radiation (PAR) of 650 μmol m^-2^ s^-1^ provided by cool white fluorescent lamps (Philips Lighting Co., Somerset, NJ). Thirty days after seeding, plants were thinned and staked, leaving four plants per pot. Granular Marathon 1% pesticide (a. i.: Imidacloprid, 1-((6-Chloro-3-pyridinyl) methyl)-N-nitro-2-imidazolidinimine) was applied to avoid sucking insect pest infestation, and plants were randomly relocated weekly within the growth chamber to avoid positional effect within the chamber. Throughout the growing season, growth chambers’ air temperature and relative humidity were monitored every 20 min with HOBO U14–001 LCD Temperature/Relative Humidity (RH) Data Logger (Onset Computer Corporation, Bourne, MA). Thick paper caps were used on the top of the HOBO data loggers to shield against direct heat radiation from the lamps. The PAR was monitored once a month with a Field Scout Light Sensor (Spectrum Technologies, Inc., Plainfield, IL).

At anthesis, two plants per pot were randomly selected and main stems were tagged. Ten days after anthesis, the temperature treatment was applied by moving one pot of each genotype from the OT regime (22/14°C day/night) to one of the growth chambers’ set at HT of 34/26°C day/night, ~85% humidity, 16 h photoperiod, and PAR of 650 μmole m^-2^ s^-1^. The dates of movement (transfer) of pots to the HT chamber were different because of different anthesis dates. Another pot of each genotype remained at OT regime throughout the experiment and served as a control. The growth chambers’ air temperature and relative humidity at HT were also constantly monitored with HOBO loggers with thick paper cap throughout the experiment period. The stress period was 16 d, after which pots were moved back to their original growth chambers. To avoid water stress, plants in both temperature regimes were fully irrigated by keeping pots in a tray filled with ~2 cm water until physiological maturity. We recognized, however, that leaving plants sitting in water for so long may cause water—logging/anoxia; which should be documented or avoided in the future studies. Once a week until physiological maturity, plants were fertigated with Miracle-Gro water-soluble fertilizer (N:P_2_O_5_:K_2_O = 24:8:16; Scotts Miracle-Gro Products, Inc., Marysville, OH) per manufacturer’s instruction. The daytime and nighttime temperature of all growth chambers were held for 12 and 8 h, respectively, with a 2-h transition period between them. During transition period, the rate of change in chamber temperature was set at 1°C/15 minutes.

### Data Collection

The date of anthesis was recorded when 50% of plants in a pot reached Feekes growth stage 10.5.1; and the days to anthesis was calculated by subtracting planting date from anthesis date. The physiological traits were measured at midday (1100 to 1300 h) on attached, fully expanded flag leaves of primary tillers of two tagged plants at an interval of four days from the second through fourteenth days after start of treatment (DAST). Leaf samples for estimating ROS and SOD activity were collected at 10 DAST from the flag leaf of a secondary tiller of each tagged plant. The yield and yield components were recorded at harvest from both tagged plants. The other two untagged plants were not used in data collection and analysis and were harvested separately and discarded.

### Leaf Temperature, Leaf Chlorophyll Content, and Chlorophyll Fluorescence

Leaf temperature was estimated by capturing an infrared (IR) image of flag leaves of primary tillers of two tagged plants with a FLIR BCAM SD (forward-looking infrared building camera, secure digital; FLIR Systems Inc., Wilsonville, OR) and processed with QuickReport 1.2 software (FLIR Systems Inc., 2009). A self-calibrating soil plant analysis development (SPAD) chlorophyll meter (SPAD-502, Spectrum Technologies, Plainfield, IL) was used to measure chlorophyll from three spots at the middle portion of the tagged flag leaf; average readings were noted. Chlorophyll fluorescence parameters were measured from dark-adapted (45 minutes) tagged flag leaves with a pulse-modulated chlorophyll fluorometer (Model OS-30P, OptiScience, Hudson, NH). Maximum quantum yield of PSII (Fv/Fm) was calculated as a ratio of variable fluorescence (Fv, a difference between maximum and minimum fluorescence) to maximum fluorescence (Fm) [38].

### Superoxide Dismutase and Superoxide Anions

Leaf samples (flag leaf) collected from the secondary tiller of a tagged plant at 10 DAST were immediately frozen in liquid nitrogen and stored at—80°C until further analysis. Superoxide dismutase activity was assayed by measuring its ability to inhibit the photochemical reduction of nitroblue tetrazolium (NBT) at 560 nm following the protocol of [39] with slight modification. Frozen tissues were ground in a cold mortar and pestle using liquid nitrogen and suspended in 1.5 ml of ice-cold extraction buffer solution containing 50 mM Na-PO_4_ buffer at pH 7.8, 1 mM EDTA.Na_2_ and 2% (w/v) polyvinypyrrolidone. The suspension was centrifuged at 20000 × g at 4°C for 20 min. The supernatant was used to measure the enzyme activity. A reaction mixture of 3 ml containing 50 mM Na-PO_4_ buffer at pH 7.8, 0.66 mM of EDTA.Na_2_, 10 mM of L-methionine, 33 μM of nitroblue tetrazolium and 0.0033 mM of riboflavin, and 50 μL of extraction buffer was added to the 150 μl of supernatant, and the tubes were illuminated with luminescent lamps for 10 min. The extinction was then read against a blank at 560 nm using a U-2000 double-beam UV/Vis spectrophotometer (Hitachi, Tokyo, Japan). One enzyme unit of SOD was defined as the amount required to inhibit photochemical reduction of NBT by 50% and was expressed as enzyme unit mg protein^-1^.

The concentration of superoxide anion was estimated according to Chaitanya and Naithani [40]. About 0.2 g of leaf samples were suspended in ice-cold 0.2 M sodium phosphate buffer at pH 7.2 containing diethyl dithiocarbamate (10^-3^ M). The suspension was immediately centrifuged at 3000 × g at 4°C for 1 min. In 200 μl of supernatant, 2 mL of nitroblue tetrazolium (2.5 × 10^-4^ M) was added and the ODA was recorded against a blank at 540 nm every 30 s for 150 s using a Hitachi U-2000 double-beam UV/Vis spectrophotometer. The superoxide anion was expressed as change in optical density min^-1^ g^-1^ fresh weight.

### Yield and Yield Components

Plants were harvested by cutting at ground level. Spikes were counted, detached, and dried to constant weight in a 38°C incubator. The remaining aboveground biomass was oven-dried at 65°C to a constant weight. The dried spikes were hand-threshed. The spikes from primary tillers of two tagged plants were used to determine the number of grains per spike and grain weight per spike. Individual grain weight was estimated by dividing grain weight per spike by number of grains per spike and expressed in mg. Yield per plant was the mean grain weight in g from all the spikes of tagged plants.

### Heat Susceptibility Index (HSI)

The heat susceptibility index (HSI) for grain yield was calculated according to Fischer and Maurer [41]:

HSI=(1−Y/Yp)/D

where Y is the average grain yield per plant of an accession at HT of 34/26°C, Yp is the average grain yield per plant of the same accessions at OT of 22/14°C, and D is the stress intensity, equal to 1—X/Xp, in which X is the mean Y of all accessions and Xp is the mean Yp of all accessions. The genotypes were classified as highly tolerant (HSI ≤ 0.5), tolerant (0.5 < HSI ≤ 0.75), moderately tolerant (0.75 < HSI ≤ 1.0), and highly susceptible. Similarly, HSIs for physiological and yield traits were also calculated and used in path coefficients to find relationships among heat tolerance of various traits including yield. They were also used to rank the genotypes for tolerance to HT stress.

### Statistical Analyses

The experimental design was a split plot with three replications (three growth chambers under each temperature regime). The temperature treatment was randomly assigned to the growth chambers and considered as the main plot. The sub-plot comprised genotypes. SAS 9.2, PROC GLIMMIX (SAS Institute Inc., Cary, NC, 2003), was used to analyze the data with block, temperature, and genotypes as class variables; block and block × temperature as random effects; and all other variables as fixed effects [42]. The least square means were separated at p < 0.05 after Tukey-Kramer adjustment. The time series data on leaf chlorophyll, flag leaf temperature, and Fv/Fm were analyzed using the REPEATED statement and Type = CS, a covariance structure of compound-symmetry type. PROC REG in SAS was used to determine the relationship between two variables, and PROC CALIS in SAS was used to conduct path coefficient analysis. At the end, genotypes were ranked on the bases of heat susceptibility indices of physiological traits, yield, and yield components using the RANK function in Microsoft Excel (2010).

## Results

The average day/night temperature recorded during the treatment period was 33.7±0.7/26.1±0.5°C in growth chambers set for the HT regime and 21.4±0.3/14.3±0.5°C in growth chambers set for the OT regime. As expected, there was no significant effect of temperature and interaction effect of temperature × genotype on days to anthesis; however, genotypes varied for this trait. The days to anthesis ranged from 88 in TA5599 to 114 in TA5088; and the mean, median, and mode were 98, 97, and 96 respectively. There were significant effects of temperature, genotype, and temperature × genotype interaction on physiological and yield traits, unless stated otherwise (Table 2).

**Table 2:** Degrees of freedom (df) and F-values for physiological, phenological, and yield traits.

Traits	df	F-values		
	(T× G)	Temperature (T)	Genotype (G)	T × G
Leaf temperature,°C	19	2610.05 ***	11.06 ***	3.16 ***
Flag leaf chlorophyll, SPAD unit	19	147.5 **	10.67 ***	3.43 ***
Maximum quantum yield of PS II (Fv/Fm), unitless	19	289.21 ***	4.14 ***	3.57***
Superoxide dismutase (SOD), enzyme unit mg protein^-1†^	18	14.68 ***	7.03 ***	3.83 ***
Superoxide anion, Δ optical density min^-1^ g^-1^ fresh weight	19	5.5 *	1.18 ^NS^	1.42 ^NS^
Days to anthesis	19	0.18 ^NS^	17.47***	0.53 ^NS^
Spike number per plant	19	0.36 ^NS^	5.12 ***	0.65 ^NS^
Grain number per spike	19	0.09 ^NS^	47.11 ***	1.61 ^NS^
Individual grain weight, mg	19	861.18 ***	17.38 ***	3.55 ***
Grain yield, g plant^-1^	19	54.12 **	10.54 ***	2.19 **

*, **, and *** denotes significance at 0.05, 0.01, and 0.001 probability levels, respectively.

^NS^, non-significant at the 0.05 probability level.

### Leaf Temperature, Leaf Chlorophyll Content, and Chlorophyll Fluorescence

**Fig 1:**
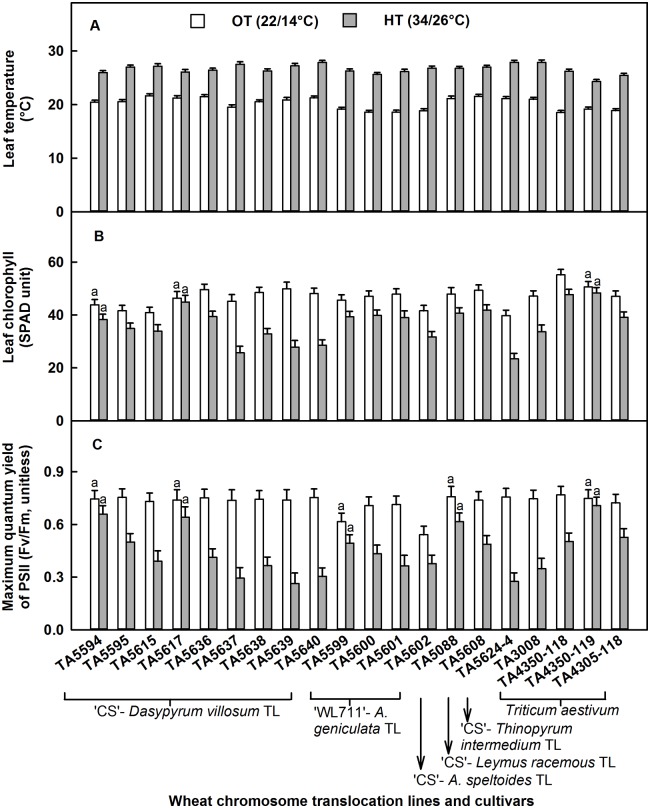
Effects of high temperature (HT) stress (34/26°C) on physiological traits. Effects of HT on (A) leaf temperature (°C), (B) leaf chlorophyll (SPAD unit), and (C) maximum quantum yield of PSII (Fv/Fm, unitless) of wheat chromosome translocation lines and cultivars are presented compared to their performance at optimum temperature (22/14°C). Each bar represents LSMeans ± standard error of LSMeans of 24 observations (2 plants × 3 replications × 4 d). Genotypes with non-significant treatment effects are indicated by the same letters on top of the bars.

The temperature regime caused significant differences in leaf temperature (p = 0.001) and leaf chlorophyll (p < 0.01). High temperature stress (34/26°C) at the grain filling stage increased leaf temperature by 6°C (Fig. 1A) and decreased leaf chlorophyll (SPAD unit) by 22% (Fig. 1B) as compared to OT (22/14°C) when averaged across the genotypes and over the first 14 d of readings. Genotypes behaved differentially according to temperature treatment for both traits at p < 0.001 (Table 2). As a consequence of HT stress, when averaged across the days and compared with OT, leaf temperature increased by > 7.5°C in TA5601, TA4350–118, TA5602, and TA5637 and by ≤ 5.5°C in TA5617, TA5636, TA4350–119, TA5608, TA5615, and TA5594. For leaf chlorophyll content, HT stress had no effect on TA5617, TA4350–119, and TA5594, but leaf chlorophyll declined by about 15% in TA4350–118, TA5599, TA5088, TA5608, and TA5600 and by 41–44% in TA5640, TA5624–4, TA5637, and TA5639.

**Fig 2:**
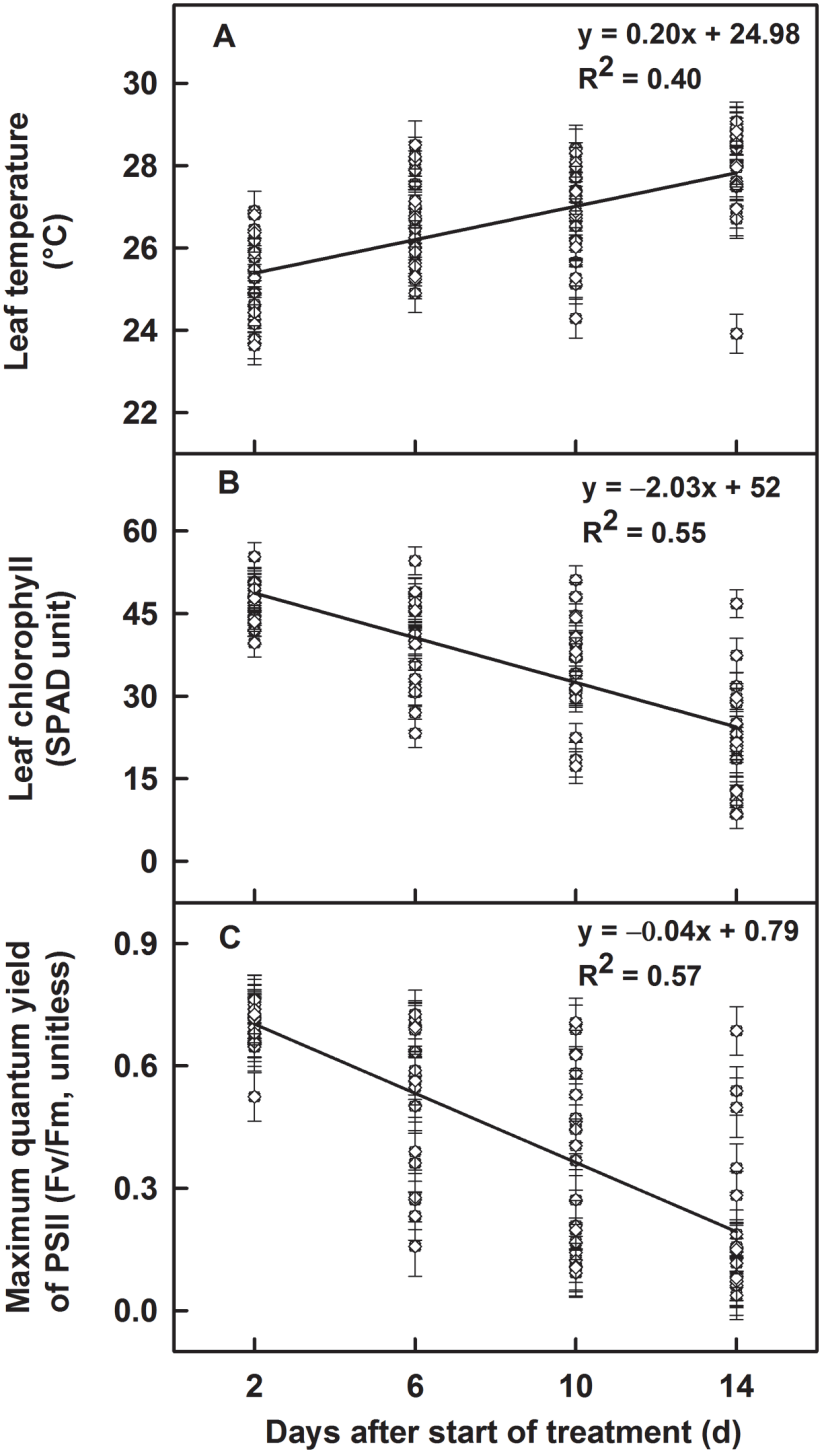
Physiological traits as a function of days after temperature treatment (DAST) under HT stress. (A) Leaf temperature (°C), (B) leaf chlorophyll (SPAD unit), and (C) maximum quantum yield of PSII (Fv/Fm, unitless) of wheat chromosome translocation lines and cultivars at high temperature stress (34/26°C) are presented as a function of DAST. Each point represents LSMeans ± standard error of LSMeans of six observations (2 plants × 3 replications).

Leaf temperature and leaf chlorophyll as a function of DAST are shown in Fig. 2. The relationship between leaf temperature and DAST under HT stress was significant and positive, with a slope of 0.2°C when averaged across the genotypes (Fig. 2A), and the relationship between leaf chlorophyll and DAST under HT stress was significant and negative, with a slope of—2.03 SPAD unit (Fig. 2B). At OT, the relationship was either not significant (leaf temperature: slope = 0.02°C, R^2^ = 0.007, and p = 0.46) or very weak (leaf chlorophyll: slope = -0.3 SPAD unit, R^2^ = 0.10, p = 0.003); therefore, OT data are not presented or discussed further. An analysis of genotypic behavior at HT stress showed that the change in leaf temperature over the time period was not significant in TA4350–119, TA5594, or TA3008; however, TA5617, TA4350–118, and TA5639 had the highest rate of increase in leaf temperature (slope = 0.32 to 0.34°C; Table 3). Similarly, under HT stress, the rate of decrease in leaf chlorophyll content throughout the experiment was < 1 SPAD unit in TA4350–119, TA5594, and TA5617; between 1 and 2 in TA5088, TA5608, TA5636, TA4305–118, and TA5595; and >3 in TA5638 and TA5639.

**Table 3:** The parameter estimates of regression equations between physiological traits and days after start of treatment (DAST) under HT stress.

Line/cultivar	Leaf temperature (°C)				Fv/Fm			
	β_0_ ^†^	β _1_ ^‡^	*Pval*	R^2^	β _0_	β _1_	*Pval*	R^2^
TA5594	25.6	0.05	0.547	0.02	0.76	–0.01	0.036	0.18
TA5595	25.2	0.23	<.001	0.53	0.85	–0.04	<0.001	0.69
TA5615	25.0	0.27	0.011	0.38	0.79	–0.05	0.004	0.47
TA5617	23.6	0.32	0.000	0.66	0.8	–0.02	0.002	0.51
TA5636	24.8	0.21	0.002	0.35	0.64	–0.03	0.039	0.18
TA5637	25.6	0.24	<0.001	0.76	0.68	–0.05	0.000	0.64
TA5638	24.7	0.20	<0.001	0.63	0.85	–0.06	<0.001	0.83
TA5639	24.5	0.34	<0.001	0.69	0.64	–0.05	00.000	0.62
TA5640	26.9	0.12	0.003	0.33	0.66	–0.04	<0.001	0.56
TA5599	24.6	0.21	0.003	0.34	0.91	–0.05	<0.001	0.74
TA5600	24.5	0.14	0.061	0.15	0.83	–0.05	<0.001	0.63
TA5601	24.3	0.24	0.010	0.39	0.86	–0.06	<0.001	0.83
TA5602	24.5	0.28	0.000	0.44	0.78	–0.05	<0.001	0.74
TA5088	25.2	0.20	0.013	0.25	0.88	–0.03	0.000	0.49
TA5608	25.8	0.15	0.007	0.29	0.89	–0.05	<0.001	0.69
TA5624–4	26.3	0.20	0.000	0.46	0.66	–0.05	<0.001	0.60
TA3008	26.9	0.12	0.092	0.19	0.77	–0.05	<0.001	0.72
TA4350–118	23.7	0.32	<0.001	0.75	0.9	–0.05	<0.001	0.63
TA4350–119	24.6	–0.03	0.485	0.02	0.74	0.00	0.014	0.25
TA4305–118	23.3	0.27	0.000	0.44	0.85	–0.04	0.001	0.43
	**Leaf chlorophyll (SPAD unit)**							
TA5594	44.5	–0.78	0.001	0.41				
TA5595	50.1	–1.90	<0.001	0.58				
TA5615	51.1	–2.17	0.002	0.51				
TA5617	53.0	–0.97	0.008	0.41				
TA5636	54.1	–1.84	0.000	0.50				
TA5637	48.5	–2.81	<0.001	0.73				
TA5638	57.2	–3.04	<0.001	0.87				
TA5639	53.0	–3.11	<0.001	0.81				
TA5640	48.5	–2.49	<0.001	0.69				
TA5599	58.6	–2.41	<0.001	0.66				
TA5600	59.5	–2.46	<0.001	0.78				
TA5601	55.0	–2.01	<0.001	0.77				
TA5602	51.8	–2.51	<0.001	0.66				
TA5088	54.6	–1.74	0.000	0.48				
TA5608	56.1	–1.79	<0.001	0.69				
TA5624–4	50.2	–2.35	<0.001	0.46				
TA3008	49.9	–2.06	<0.001	0.81				
TA4350–118	54.2	–2.01	<0.001	0.41				
TA4350–119	63.7	–0.23	<0.000	0.69				
TA4305–118	42.3	–1.89	0.001	0.84				

^†^β _0_, intercept;

^‡^β _1_, slope.

The difference between temperature regimes for maximum quantum yield of PSII (Fv/Fm, unitless) was significant at p < 0.001. High temperature stress at the grain filling stage decreased Fv/Fm by 38% (Fig. 1C) compared to OT when averaged across the genotypes and over the first 14 d of readings. Genotypes behaved differently according to the temperature treatment at p < 0.001 (Table 2). When averaged across the days, HT stress had no effect on TA4350–119, TA5594, TA5617, TA5088, and TA5599 for Fv/Fm compared with OT. However, Fv/Fm decreased by < 35% in TA5608, TA5595, TA5602, and TA4305–118 and by 60–64% in TA5640, TA5624–4, and TA5639. Fv/Fm as a function of DAST is shown in Fig. 2. A significant negative relationship was evident between Fv/Fm and DAST under HT stress when averaged across the genotypes, with a slope of—0.04 unit (Fig. 2C). At OT, the relationship of Fv/Fm with DAST was very weak (slope = -0.005 unit, R^2^ = 0.08); therefore, data are not presented or discussed. Further analysis of genotypic behavior at HT stress showed that TA4350–119, TA5594, and TA5617 had the lowest rate of decrease in Fv/Fm (slope < -0.02 unit), and TA5638 and TA5601 had the highest rate of decrease (slope > -0.06 unit) during the time period (Table 3).

**Fig 3:**
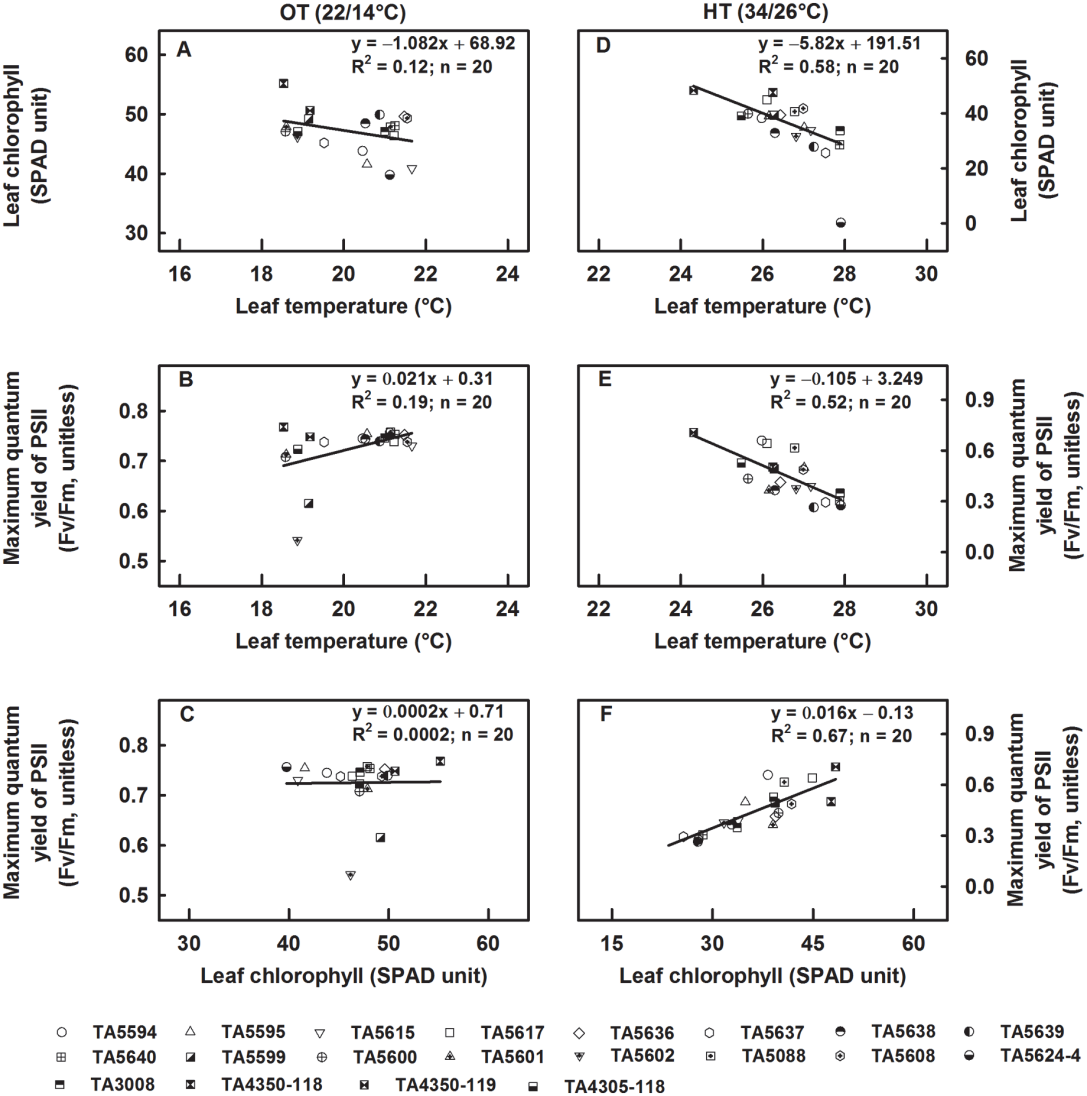
Relationships among physiological traits at two temperature regimes. At optimum temperature (22/14°C) (A, B, and C) and at high temperature (34/26°C) (D, E, F).

Relationships among leaf temperature, leaf chlorophyll, and Fv/Fm are presented in Fig. 3. At OT, there was no relationship between leaf chlorophyll and leaf temperature (R^2^ = 0.12, p > 0.05) (Fig. 3A), and a very weak positive relationship between Fv/Fm and leaf temperature (R^2^ = 0.19, p = 0.05) (Fig. 3B). The relationship between Fv/Fm and leaf chlorophyll content was non-significant (R^2^ = 0.0002, p > 0.05) (Fig. 3C). At HT, there was a highly negative relationship between leaf chlorophyll and leaf temperature (R^2^ = 0.58, p < 0.001) (Fig. 3D); and Fv/Fm and leaf temperature (R^2^ = 0.52, p < 0.001) (Fig. 3E). The relationship between Fv/Fm and leaf chlorophyll at HT stress was strongly positive (R^2^ = 0.67, p < 0.001) (Fig. 3F).

### Superoxide Dismutase (SOD) and Superoxide Anion

There was a significant difference between temperature regimes for SOD activities and concentration of the superoxide anion, a ROS. High temperature stress decreased SOD activity by 8% and increased superoxide anion concentration by 44% (0.12 changes in OD min^-1^ g^-1^ FW) compared to OT when averaged across the genotypes. Genotypes behaved differentially according to the temperature treatment for SOD at p < 0.001 (Table 4). The effect of HT stress was not evident in 17 genotypes for SOD, but SOD activity decreased by 29% in TA5638 and TA5600 and by 52% in TA4305–118. Genotype and temperature × genotype interaction was not significant for the superoxide anion, so data for this trait were not presented in further detail (p > 0.05) (Table 2).

**Table 4:** Effects of high temperature stress on grain number per spike and superoxide dismutase of wheat chromosome translocation lines and cultivars^†^.

**Line/cultivar**	**Grain number per spike**				**SOD(enzyme unit per mg protein)**			
	**OT**	**HT**	**Mean**		**OT**		**HT**	
TA5594	69.8	73.8	69.8	ABC	1.72		2.05	
TA5595	65.0	77.0	65.0	ABC	1.83		1.76	
TA5615	51.0	64.1	51.0	CD	1.95		2.21	
TA5617	52.7	46.5	52.7	DE	2.00		1.95	
TA5636	58.7	65.0	58.7	BCD	1.95		1.77	
TA5637	59.2	65.8	59.2	BCD	-^‡^		-	
TA5638	82.0	79.5	82.0	A	1.93	a	1.37	b
TA5639	53.9	59.5	53.9	CD	1.88		1.80	
TA5640	64.5	58.5	64.5	BCD	1.45		1.10	
TA5599	36.7	32.3	36.7	EF	2.20		1.82	
TA5600	23.0	28.3	23.0	FG	2.66	a	1.89	b
TA5601	17.5	18.9	17.5	FG	2.28		2.06	
TA5602	28.5	16.5	28.5	FG	2.05		2.26	
TA5088	74.6	75.7	74.6	ABC	1.89		1.65	
TA5608	36.2	35.8	36.2	EF	1.88		2.09	
TA5624–4	25.5	23.1	25.5	FG	2.20		2.06	
TA3008	66.8	58.2	66.8	BCD	2.02		1.68	
TA4350–118	58.5	67.5	58.5	BCD	2.29		2.31	
TA4350–119	37.2	25.5	37.2	FG	2.00		2.23	
TA4305–118	29.0	33.0	29.0	FG	2.19	a	1.06	b
Mean	49.5	50.2			2.02	a	1.85	b

^†^Within columns, significant different at p ≤ 0.05 according to Tukey-Kramer test is indicated by a different capital letter; within rows, it is indicated by a different small letter. OT = optimum temperature (22/14°C); HT = high temperature (34/26°C); SOD, super oxide dismutase;

^‡^Missing sample was indicated by ‘-’.

### Spike Number, Grain Number, Individual Grain Weight, and Grain Yield

Effects of temperature and interaction of temperature × genotype were not significant for spike number per plant and grain number per spike; however, a significant variation among genotypes was evident at p < 0.001 (Table 2). The average spike number per plant ranged from 1.2 in TA5088 to 2.9 in TA5608; and grain number per spike ranged from below 33 in TA5600, TA5601, TA5602, TA5624–4, TA4350–119, and TA4305–118 to more than 66 in TA5594, TA5595, TA5638, and TA5088.

**Fig 4:**
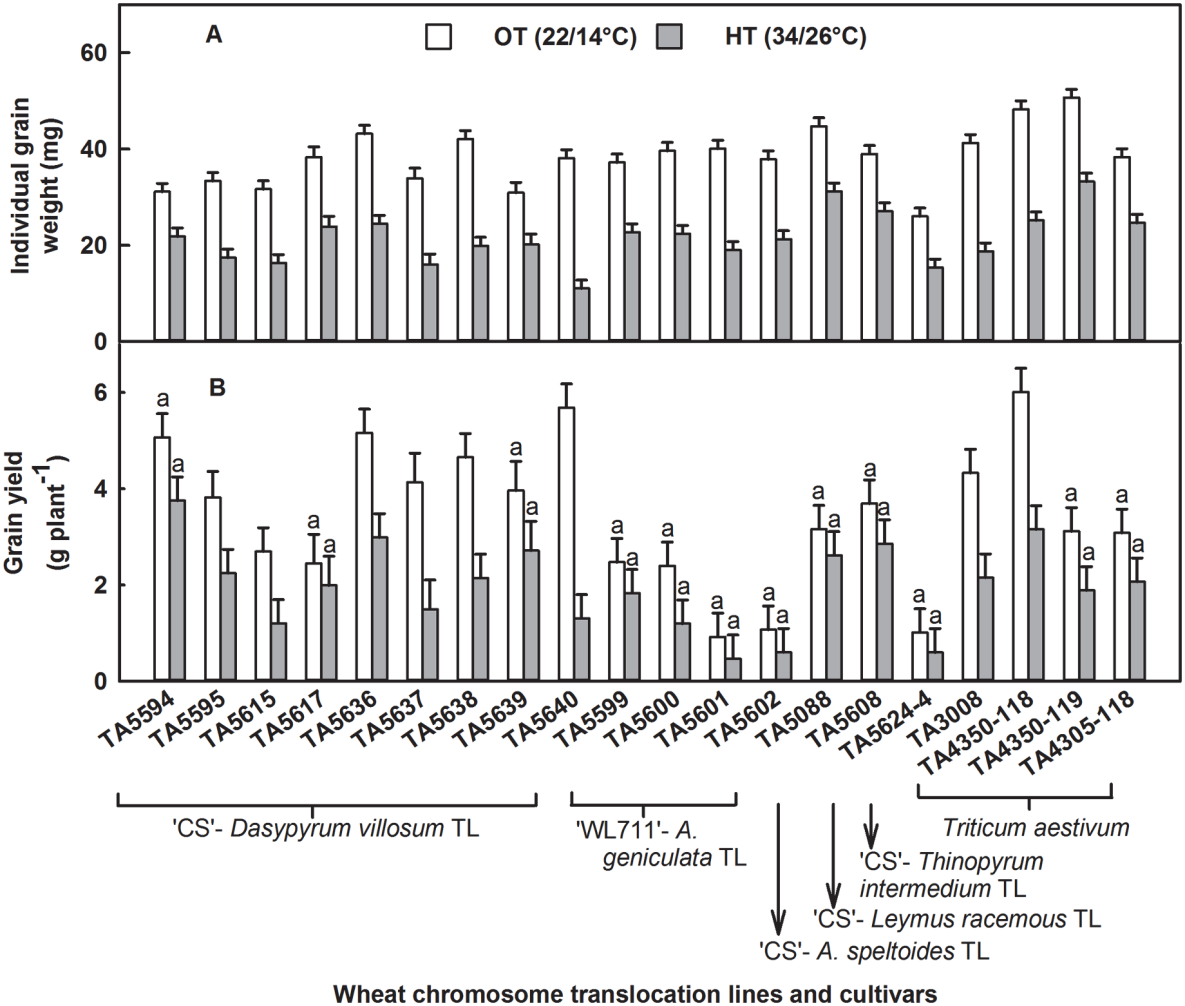
Effects of high temperature (HT) stress (34/26°C) on individual grain weight (mg) and grain yield (g per plant). Effects of HT stress on (A) individual grain weight and (B) grain yield of wheat chromosome translocation lines and cultivars are presented compared with their performance at optimum temperature. Each bar represents LSMeans ± standard error of LSMeans of 6 observations (2 plants × 3 replications). Genotypes with non-significant treatment effects are indicated by the same letters on the top of the bars.

There was a significant difference between temperature regimes for IGW and grain yield per plant, and genotypes behaved differently according to temperature treatment for these traits at p ≤ 0.004 (Table 2). High temperature stress decreased IGW and grain yield by ~43% compared with OT when averaged across the genotypes. As a result of HT, IGW decreased by 30% in TA5594, TA5088, and TA5608 and by 71% in TA5640 (Fig. 4A). High temperature stress significantly decreased grain yield in TA5595, TA5615, TA5636, TA5637, TA5638, TA5640, TA3008, and TA4350–118, and the decrease ranged from about 41% in TA5595 to 77% in TA5640. The decrease in grain yield because of HT stress was not statistically significant in other genotypes (Fig. 4B).

### Heat Susceptibility Index for Physiological, Yield and Yield Traits

**Fig 5:**
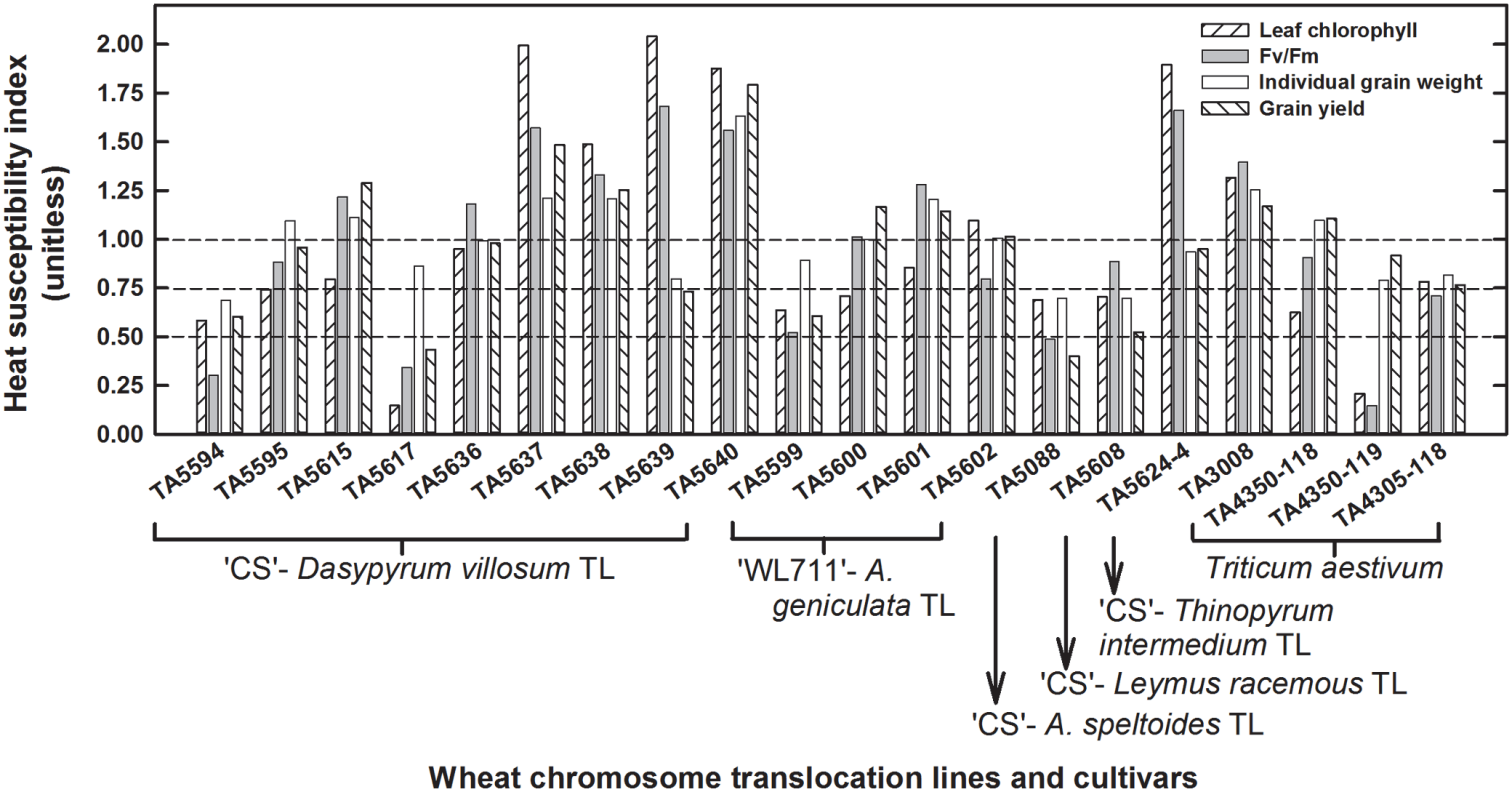
Heat susceptibility index (HSI) for physiological, yield and yield traits of wheat chromosome translocation lines and cultivars. The horizontal lines classified genotypes into highly tolerant (HSI ≤ 0.5), tolerant (0.5 < HSI ≤ 0.75), moderately tolerant (0.75 < HSI ≤ 1.0), and highly susceptible (> 1.0) to high temperature stress.

Heat susceptibility indices for leaf chlorophyll, maximum quantum yield of PSII (Fv/Fm), individual grain weight and grain yield are presented in Fig. 5. The genotypes TA5617 and TA4350–119 had ≤ 0.5 HSI for leaf chlorophyll; and TA5594, TA5599, TA5088, TA5608, TA4350–118 had HSI between 0.5 and 0.75. The genotypes TA5594, TA5617, TA5088, TA4350–119 had ≤ 0.5 HSI for Fv/Fm; and TA5599, TA4305–118 had HSI between 0.5 and 0.75. The genotypes TA5594, TA5088, TA5608 had ≤ 0.75 HSI for individual grain weight and all other genotypes had > 0.75 HSI. The genotypes TA5617 and TA5088 had < 0.5 HSI for grain yield, and TA5594, TA5639, TA5599, TA5608 had HSI between 0.5 and 0.75. The genotypes TTA5637, TA5638, TA5640, and TA3008 had > 1.0 HSI for all traits.

### Pearson’s Correlation Coefficients and Path Coefficient Analysis

At OT, there was no significant correlation between individual grain weight and grain yield per plant; and the maximum quantum yield of PSII (Fv/Fm) had no significant correlation with leaf chlorophyll and individual grain weight. A significant positive correlation was evident between Fv/Fm and grain yield per plant; and leaf chlorophyll had significant positive correlation with individual grain weight and grain yield per plant (Table 5). At HT, there was significant positive correlation among Fv/Fm, individual grain weight and grain yield per plant. Although leaf chlorophyll had strong positive correlation with Fv/Fm and individual grain weight, there was no correlation with grain yield per plant.

**Table 5:** Pearson correlation coefficients between yield, yield components, and physiological traits with significant G × T interactions. Number of data point used (n) = genotypic mean = 20.

	**OT** ^†^ **(22/14°C)**			**HT (34/26°C)**		
**Traits**	**Grain yieldper plant**	**Individualgrain weight**	**Leaf Chlorophyll(SPAD Unit)**	**Grain yieldper plant**	**Individualgrain weight**	**Leaf Chlorophyll (SPAD Unit)**
**Grain yield per plant**	1.00			1.00		
**Individual grain weight**	0.27^NS^	1.00		0.45*	1.00	
**Leaf chlorophyll (SPAD unit)**	0.53*	0.75***	1.00	0.41^NS^	0.80***	1.00
**Fv/Fm**	0.53*	0.08^NS^	0.34^NS^	0.47*	0.76***	0.82***

^†^OT, optimum temperature; HT, high temperature. Fv/Fm, maximum quantum yield of PSII;

*, **, *** Significant at the 0.05, 0.01, and 0.001 probability level, respectively.

^NS^, non-significant at the 0.05 probability level.

**Fig 6:**
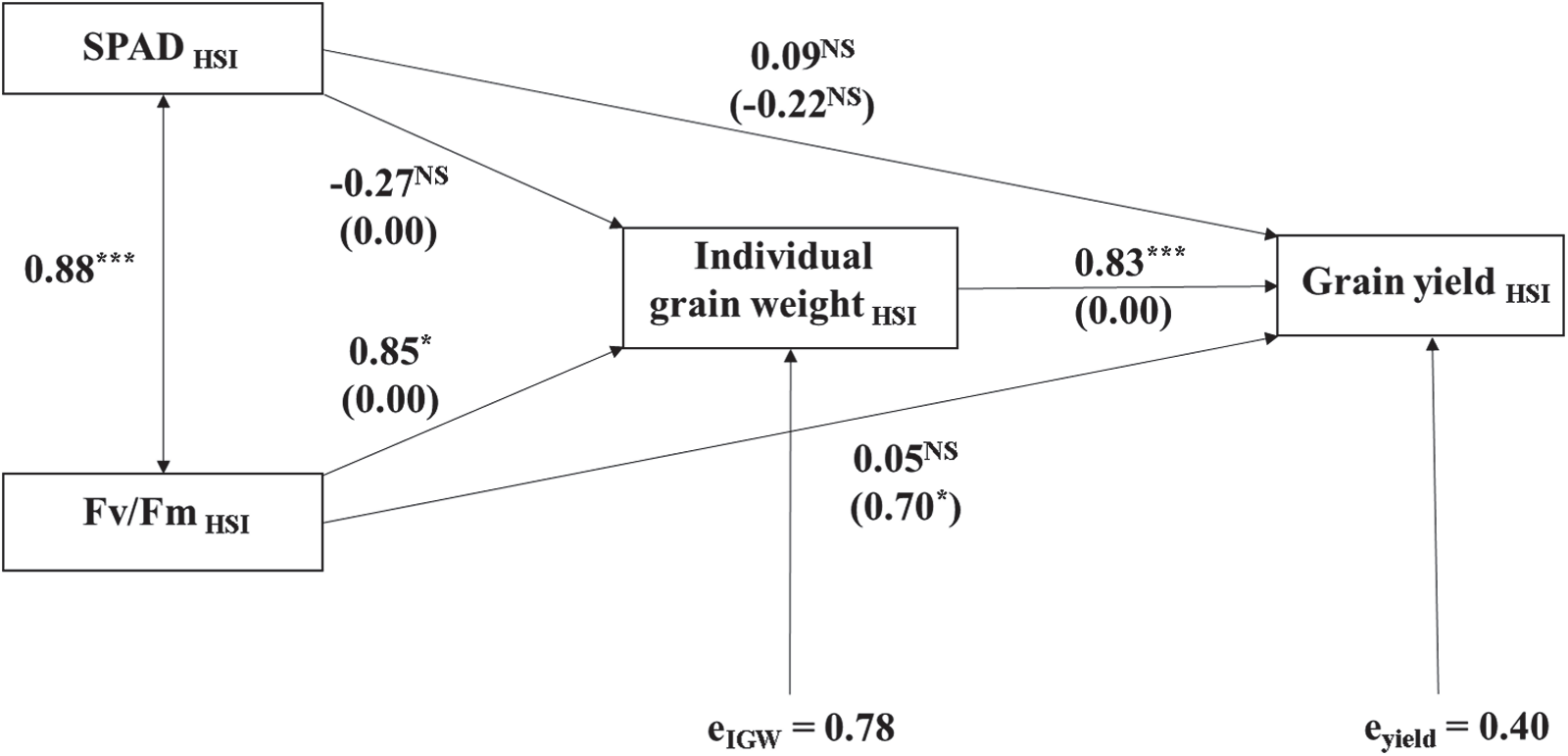
Path diagram indicating direct and indirect effects between heat susceptibility indices of physiological and yield traits and to HSI of grain yield. The value besides two way arrow indicates Pearson correlation coefficient. The values beside one way arrow indicate direct effects and those in parenthesis indicate indirect effects. *, **, and *** denotes significance at 0.05, 0.01, and 0.001 probability levels, respectively. e_IGW_, and e_yield_ denotes error term for individual grain weight and grain yield, respectively. ^NS^, non-significant.

A path coefficient analysis performed to identify correlation or direct and indirect effects of heat responses and relationships of heat susceptibility i.e. heat susceptibility indices (HSIs) of physiological and yield traits to each other and to HSI of yield are presented in Fig. 6. There was a strong positive correlation between SPAD_HSI_ and Fv/Fm_HSI_. The SPAD_HSI_ had neither direct nor indirect effect on IGW_HSI_ and Yield_HSI_; however, the Fv/Fm_HSI_ had significant direct effect on IGW_HSI_ and indirect effect on Yiled_HSI_. The IGW_HSI_ had a strong direct effect on Yield_HSI_.

### Ranking of Genotypes Based on HSIs of Physiological, Yield Traits, and Yield

The rankings of genotypes on the bases of their response to HT stress for traits i.e. Fv/Fm_HSI_, IGW_HSI_, and Yield_HSI_ are presented in Table 6. There was evidence of direct and indirect effects of these traits on Yield_HSI_ (Fig. 6). On the basis of final ranking estimated from a mean of all rankings, TA5594, TA5617, and TA5088 were the top three genotypes tolerant to HT stress; and TA5640 and TA5637 were highly susceptible genotypes with rankings of 19 and 20.

**Table 6:** Ranking of wheat chromosome translocation lines and cultivars on the basis of their response to HT stress (HSIs) for maximum quantum yield of PSII (Fv/Fm), individual grain weight (IGW), and grain yield.

**Line/cultivar**	**Fv/Fm**_**HSI**_ ^†^ **(unitless)**		**IGW**_**HSI**_ **(unitless)**		**Grain yield**_**HSI**_ **(unitless)**		**Mean of ranks**	**Final rank**
	Value	Rank	Value	Rank	Value	Rank		
TA5594	0.30	2	0.69	1	0.60	4	2.3	1
TA5595	0.88	8	1.09	13	0.96	10	10.3	8
TA5615	1.22	13	1.11	15	1.29	18	15.3	16
TA5617	0.34	3	0.86	7	0.43	2	4.0	3
TA5636	1.18	12	0.99	10	0.98	11	11.0	11
TA5637	1.57	18	1.21	18	1.48	19	18.3	19
TA5638	1.33	15	1.21	17	1.25	17	16.3	17
TA5639	1.68	20	0.80	5	0.73	6	10.3	8
TA5640	1.56	17	1.63	20	1.79	20	19.0	20
TA5599	0.52	5	0.89	8	0.61	5	6.0	6
TA5600	1.01	11	1.00	11	1.17	15	12.3	12
TA5601	1.28	14	1.21	16	1.14	14	14.7	15
TA5602	0.80	7	1.01	12	1.01	12	10.3	8
TA5088	0.49	4	0.70	2	0.40	1	2.3	1
TA5608	0.89	9	0.70	3	0.52	3	5.0	5
TA5624–4	1.66	19	0.94	9	0.95	9	12.3	12
TA3008	1.39	16	1.25	19	1.17	16	17.0	18
TA4350–118	0.90	10	1.10	14	1.11	13	12.3	12
TA4350–119	0.15	1	0.79	4	0.92	8	4.3	4
TA4305–118	0.71	6	0.82	6	0.77	7	6.3	7

^†^HSI, heat susceptibility index.

## Discussion

We evaluated 16 wheat chromosome translocation lines that had a small chromosome segment from different wild wheat species introgressed into spring wheat cultivars for HT stress tolerance at the grain filling stage. The effect of HT stress was highly significant for all physiological and yield traits except grain number per spike, and we demonstrated genotypic variability in chromosome translocation lines for physiological traits, yield components, and grain yield. This information can be used in breeding programs to develop stress-tolerant cultivars.

Canopy temperature shows the efficiency with which evaporative cooling is occurring from the plant surface, and it has been used to estimate heat stress in field crops under fully or limited irrigated conditions [16,43–45]. Ayeneh et al. [46] showed a strong positive correlation between canopy temperature and flag leaf temperature (r = 0.91) in 13 wheat genotypes grown under HT stress. In this study, the HT stress at the grain filling stage increased flag leaf temperature by 6°C as compared to OT (Fig. 1A); and there was genotypic variation in flag leaf temperature. Genotypic variation in flag leaf temperature under HT stress was evident in wild wheat relatives [1]; and the variability in flag leaf temperature at HT stress might be attributed to differential decrease in vascular capacity and impairment of cooling mechanisms[47,48].

High temperature stress increased superoxide anions by 44% and decreased SOD activity by 8% (Table 4). Reactive oxygen species like superoxide anions are a product of regular sub-cellular processes and are strictly regulated by the antioxidant enzyme SOD to avoid their detrimental effects on cellular structures. High temperature stress increases the formation of ROS in chloroplast owing to the over excitation of chlorophyll molecules and decreases in antioxidant enzyme activity [13,17]. High temperature stress decreased leaf chlorophyll and maximum quantum yield of PSII (Fig. 1B, and 1C). Genotypic variation for these attributes was evident. High temperature stress is well known to decrease leaf chlorophyll [1,10,13], which could be the result of thylakoid membrane damage [11,12], lipid peroxidation of chloroplast membranes [13], and inhibition of chlorophyll biosynthesis [14]. The study showed that the longer the duration of HT stress, the higher the leaf temperature but the lower the leaf chlorophyll and the maximum quantum yield of PSII (Fv/Fm) (Fig. 2). It has been reported that light is one of the primary stress parameters for PSII performance, and significant differences in Fv/Fm can be found even among upper and lower leaves of a wheat plant due to shading [49]. In this study, as the fluorescence data was collected from the upper most flag leaf only; and the light level in both HT and OT chambers were about 650 μmol m^-2^ s^-1^ provided with the same make and model of white fluorescent lamps, we assumed that the influence of light source on Fv/Fm was negligible; and difference in Fv/Fm among genotypes was due to their differential tolerance to high temperature. Genotypic variation for the rate of increase or decrease of respective physiological traits at HT stress was evident (Table 3). In addition, under OT, there were no evidence of a relationship between leaf temperature and leaf chlorophyll or Fv/Fm, and leaf chlorophyll content and Fv/Fm (Fig. 3), whereas under HT stress, the relationships were either highly negative (between leaf temperature and leaf chlorophyll or Fv/Fm) or highly positive (between leaf chlorophyll and Fv/Fm). In this study, under HT stress, for a unit increase in leaf temperature, there was an evidence of decrease in leaf chlorophyll by 5.82 SPAD unit, and Fv/Fm by 0.105 unit; and for a decrease in one SPAD unit there was an evidence of decrease in Fv/Fm by 0.016 unit (Fig. 3).

In this study, HT stress had no effect on grain number per spike (Table 4). The absence of a treatment effect on grain number in this study contrasted with earlier findings from a controlled environment experiment, where winter wheat cultivar ‘Karl 92’ had a 12.5% reduction in grain number when temperature increased from 25/20°C to 35/20°C from 10 d after anthesis [18], and from a field experiment, where Zhong-hu and Rajaram [50] observed about a 13% decrease in grain number between late- and normal-planted wheat. Hays et al. [51] reported that HT stress at 10 d after anthesis induced ethylene production, resulting in kernel abortion in heat-susceptible winter wheat ‘Karl 92’ but not in heat-tolerant spring wheat cultivar ‘Halberd’. This result showed that genotypes used in the current study were tolerant to HT stress for kernel abortion; however, we recommend further study of these genotypes to elucidate the mechanism of embryo tolerance to HT stress. High temperature stress decreased individual grain weight (Fig. 4A) by 43%, which is about 7% lower than the decline observed by Yang et al. [20] in 30 synthetic hexaploid wheat varieties and 13% higher than the decline observed by Gibson and Paulsen [18] in ‘Karl 92’ subjected to HT stress from 10 d after anthesis. The decrease in IGW under HT stress might be due to an increase in leaf senescence and decrease in grain-filling duration [8,10,20,21], and genotypic variation for this trait has been observed by other authors [15,18–20]. High temperature stress decreased grain yield per plant by 44%. Yang et al. [20] and Gibson and Paulsen [18] observed about 54 and 78% decreases, respectively, in grain yield when HT stress was applied to wheat 10 d after anthesis. Grain number per spike and IGW are two important factors of grain yield per plant. The absence of treatment effect on grain number might be the reason for the smaller decrease in grain yield in this study compared with earlier studies. As in earlier work, genotypes differed significantly in their response to HT stress for grain yield.

A correlation and path diagram analyses to investigate correlations between heat responses of physiological and yield traits (HSIs) to each other and to grain yield HSI showed that there was a strong correlation between SPAD_HSI_ and Fv/Fm_HSI_ (Fig. 6); however, SPAd_HSI_ had no significant effect on IGW_HSI_ and Yield_HSI_, whereas Fv/Fm_HSI_ had a strong correlation with IGW_HSI_ and Yield_HSI_. There was evidence that the nature of the effects of Fv/Fm_HSI_ on IGW_HSI_ and of IGW_HSI_ on Yield_HSI_ were direct but the effect of Fv/Fm_HSI_ on Yield_HSI_ was indirect. These results provide evidence that there might be strong associations (cause-effect relationships) among HSIs of Fv/Fm, IGW, and grain yield; this implies that the physiological trait such as Fv/Fm is useful in screening genotypes for HT stress tolerance. However, we are of view that gas exchange traits would have added further valuable information on the behavior of genotypes at HT; and therefore we recommend the collection of gas exchange data in future studies. On the bases of above result, we used Fv/Fm_HSI_, IGW_HSI,_ and Yield_HSI_ in ranking the genotypes for HT stress tolerance instead of using HSIs of Yield and/or IGW only. The ranking showed that TA5594, TA5617, and TA5088 were highly tolerant (ranked 1to 3), and TA5637 and TA5640 were highly susceptible (ranked 19 and 20) to HT stress compared with other lines and the check cultivars.

## Conclusions

This study revealed genetic variability among wheat chromosome translocation lines for HT stress tolerance at the grain filling stage. It illustrated that a decrease in individual grain weight was the main reason for yield decline and quantified that an increase in HT stress (high leaf temperature) adversely affected leaf chlorophyll and Fv/Fm; and a decrease in Fv/Fm might be one of the physiological reasons for subsequent decline in individual grain weight. In this study, we identified TA5594, TA5617, and TA5088, having small segment of chromosome from *Haynaldia villosa* or *Aegilops speltoides*, as highly tolerant to HT stress at grain filling stage. These tolerant genotypes may be used to improve HT stress tolerance of wheat cultivars. We recommend further screening of a larger population of translocation lines. Because other translocation lines might have chromosome segments from different wild relatives or different genomes, there are good possibilities of discovering more HT stress tolerant genotypes.
